# CYFIP family proteins between autism and intellectual disability: links with Fragile X syndrome

**DOI:** 10.3389/fncel.2014.00081

**Published:** 2014-03-27

**Authors:** Sabiha Abekhoukh, Barbara Bardoni

**Affiliations:** ^1^CNRS, Institute of Molecular and Cellular Pharmacology, UMR 7275Valbonne, France; ^2^University of Nice Sophia-AntipolisNice, France; ^3^CNRS, International Associated Laboratories–NEOGENEXValbonne, France

**Keywords:** autism, intellectual disability, Fragile X, CYFIP family proteins, WAVE complex, F-actin, dendritic spines

## Abstract

Intellectual disability (ID) and autism spectrum disorders (ASDs) have in common alterations in some brain circuits and brain abnormalities, such as synaptic transmission and dendritic spines morphology. Recent studies have indicated a differential expression for specific categories of genes as a cause for both types of disease, while an increasing number of genes is recognized to produce both disorders. An example is the Fragile X mental retardation gene 1 (*FMR1*), whose silencing causes the Fragile X syndrome, the most common form of ID and autism, also characterized by physical hallmarks. Fragile X mental retardation protein (FMRP), the protein encoded by *FMR1, *is an RNA-binding protein with an important role in translational control. Among the interactors of FMRP, CYFIP1/2 (cytoplasmic FMRP interacting protein) proteins are good candidates for ID and autism, on the bases of their genetic implication and functional properties, even if the precise functional significance of the CYFIP/FMRP interaction is not understood yet. CYFIP1 and CYFIP2 represent a link between Rac1, the WAVE (WAS protein family member) complex and FMRP, favoring the cross talk between actin polymerization and translational control.

## INTRODUCTION

Intellectual disability (ID) and autism spectrum disorders (ASD) are a serious public health problem. The causes of ID and autism are extremely heterogeneous, ranging from environmental to genetic and even combinations of the two. Autism is a disorder of neural development characterized by impaired social interaction and communication and by restricted and repetitive behavior. Signs all begin before a child is three years old. Autism is a pervasive developmental disorder (PDD) that involves severe deficits in a person’s ability to communicate and interact with others. Children with autism often have trouble using their imagination, have a limited range of interests, and may show repetitive patterns of behavior or body movements. Different people with autism can have very different symptoms, thus health care providers consider autism as a “spectrum” disorder (ASD), a group of disorders with similar features, including autistic disorder (also called “classic” autism), Asperger syndrome and PDD not otherwise specified or atypical autism. ASD is a very common disorder (prevalence of 1:1000 newborns). Worldwide, 2–3% of the population is affected by mild to severe ID. The economic and social consequences of this disorder are very important since the majority of people with ID require long-term supportive care or service. New technologies allowed the identification of many mutations in ID patients affecting single genes. Thus, genetic alterations identified in ID are fast expanding. It is interesting to underline that mutations in the same gene can cause ID or ASD or both and interestingly more than 80% of XLID (X-Linked Intellectual Disabilities) genes are also cause of autism (Schwartz and Neri, 2012; Brentani et al., 2013; Murdoch and State, 2013). These two disorders have in common alterations in some brain circuits and brain abnormalities, such as synaptic transmission and dendritic spine morphology (Gilman et al., 2011). Remarkably, even if both ID and ASD are heterogeneous in their genetic and molecular bases, recent studies have indicated a significant enrichment for specific categories of genes as a cause for both types of disease, while an increasing number of genes is recognized to produce both disorders. Search for genes causing ID and ASD, as well as characterization of animal models for these disorders, allows to better understand the physiopathology of these diseases and to understand the functioning of the brain. During the last few years huge efforts have been made by many groups in this field, that have indicated the involvement of several categories of genes in these disorders, including genes regulating axon outgrowth, synaptogenesis, cell–cell adhesion, GTPase signaling and the actin cytoskeleton (Gilman et al., 2011; Voineagu and Eapen, 2013). On the same track, also for ID common pathways seem to emerge (e.g., Rho-GTPase and other small GTPase pathways, JNK and Ras signaling), even if the full picture is in continuous evolution (Davidovic et al., 2011; Hashimoto et al., 2011; Pavlowsky et al., 2012; Melko et al., 2013). The increasing number of genes involved in ASD allowed the generation of networks of genes involved in this disease that are spatio-temporally coexpressed (Willsey et al., 2013).

An example of disorder characterized by both ID and ASD is the Fragile X syndrome (FXS), the most common form of inherited ID (estimated prevalence of 1:4000 males and 1:8000 females) and also the most frequent known cause of autism (Auerbach et al., 2011). Silencing of the *FMR1* gene, encoding the Fragile X mental retardation protein (FMRP), causes FXS. The clinical manifestations characterizing patients affected by FXS include moderate to severe cognitive impairment, elongated facial features, attention deficit, hyperactivity, stereotypy, seizure, impulsivity, sensory hyperarousal, anxiety, and autistic behavior (Bardoni et al., 2000). In brain the major phenotype of FXS patients and FXS animal models (mouse, *Drosophila*) is the presence of dendritic spines that are longer, thinner and denser than normal (Comery et al., 1997; Irwin et al., 2000, 2002; Schenck et al., 2003). They represent the cellular defect underpinning the neuronal dysfunctions characterizing this disorder. Interestingly, this morphological defect is associated to the alteration of different forms of synaptic plasticity in mouse brain (Dolen et al., 2010). FMRP is an RNA-binding protein that plays a role in several steps of mRNA metabolism and, in particular, in translational control at the synaptic level. The absence of FMRP may alter the processing, localization or translational regulation of mRNAs encoding pre- and post-synaptic proteins. These defects can account for the abnormal maturation of dendritic spines in FXS (Swanger and Bassell, 2011; Bardoni et al., 2012; Maurin et al., 2014).The lack of FMRP interferes with mechanisms underlying metabotropic glutamate receptor (mGluR) receptor-dependent long-term depression (LTD) – a prominent form of synaptic plasticity (Huber et al., 2002) and epileptogenesis (Chuang et al., 2005). Indeed, mGluR receptor-dependent LTD in the hippocampus is amplified in the absence of FMRP, whereas NMDA receptor-dependent LTD is not (Huber et al., 2002). mGlu5 receptor-dependent long-term potentiation (LTP) is instead reduced in the cerebral cortex of *Fmr1* null mice (Wilson and Cox, 2007). The mGlu5 receptor-dependent LTD found in animal models of FXS, unlike the one found in wild-type animals, is insensitive to inhibitors of protein synthesis (Bureau et al., 2008). One possibility is that the constitutive abnormality in the expression of synaptic proteins alters long-term responses to mGluR5 receptor activation in this syndrome. This data is consistent with the increased internalization of AMPAR in FMRP-deficient dendrites in the basal state (Nakamoto et al., 2007). Moreover, it is noteworthy that mGluR5 receptors are less associated to the Homer protein in the brain of *Fmr1* knockout mice, which is suggestive of an important alteration in receptor signaling (Giuffrida et al., 2005). Hippocampal epileptogenesis is another form of synaptic plasticity that depends on group I mGlu receptor activation and protein synthesis and is altered in *Fmr1 *null mice. The increased excitability in the absence of FMRP can be reversibly blocked by 2-methyl-6-(phenylethynyl)pyridine (MPEP) a specific antagonist of mGluR5, suggesting elevated constitutive mGluR5 receptor activation in FXS. *Fmr1* mutant mice with a 50% reduction in mGluR5 expression exhibited a rescued phenotype (Dolen et al., 2010). However, other different pathways are controlled by FMRP (Davidovic et al., 2011) and up to date is has been difficult to dissect signaling defects determining ID and signaling defects relevant for autistic behavior. Two main paths however, seem to emerge as a link between the two pathologies in FXS: one is represented by the correct balance of the mGluR signaling pathway (Auerbach et al., 2011) and the other by the link with RhoGTPase activity and actin remodeling, represented by the two cytoplasmic FMRP interacting proteins CYFIP1 and CYFIP2 (Schenck et al., 2001, 2003).

## CYFIP PROTEINS: THE WAVE COMPLEX AND BEYOND

**FIGURE 1:**
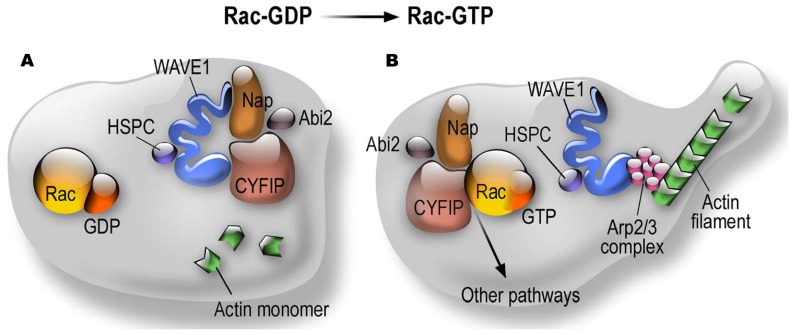
**Dynamics of CYFIP Proteins in the context of the WAVE complex and actin polymerization. (A)** Structural organization of the WRC in the Rac-GDP condition; **(B)** Structural organization of the WRC in the Rac-GTP condition.

CYFIP1 and CYFIP2 (also known as PIR121) are members of a gene family highly conserved during evolution (Schenck et al., 2001). They are components of the canonical WAVE regulatory complex (WRC) that, besides the WAVE protein (WAS protein family member), also contains the NAP1 (NCKAP1 or HEM1 in hematopoietic cells) subunit, the ABI1 protein (or one of its paralogous proteins, ABI2 or NESH) and BRK1 (also known as HSPC300) (Cory and Ridley, 2002; Derivery et al., 2009). The WAVE complex transduces Rac signaling via CYFIP1 to trigger Arp2/3-dependent actin nucleation (Cory and Ridley, 2002; Derivery et al., 2009). This process is important in the spatio-temporal regulation of actin dynamics to get correct cell migration, cell polarity (in particular in neurons the axonal polarity), cell adhesion and vesicle trafficking. The WASP family (Wiskott–Aldrich syndrome protein) is composed by five members: WASP, N-WASP and WAVE1, 2, and 3. All these proteins are characterized by the presence of a VCA (verprolin homology, central and acidic region) domain able to activate the Arp2/3 complex. Indeed, as shown in **Figure 1**, CYFIP1/2 interact with the small RhoGTPase Rac1. Upon this binding the subcomplex CYFIP1/2, Nckap1/ABI1 leaves the inactive WAVE holo complex. While WAVE can now interact with Arp2/3, the CYFIP1-containing subcomplex is available to interact with other proteins (**Figure 1**) and then also with FMRP. Indeed, we have shown that the FMRP/CYFIP interaction is GTP dependent (Schenck et al., 2001, 2003). Purified Rho GTPase Rac1 can bind and activate recombinant WAVE complex *in vitro* (Lebensohn and Kirschner, 2009) and the crystal structure of the WAVE complex identified a potential binding site for Rac1 in CYFIP1(Chen et al., 2010). Interestingly it has also been shown that WAVE complex activation is obtained by the cooperation of the Arf and Rac1 GTPases (Koronakis et al., 2011).

Consistent with this function, the WAVE complex has been shown to be involved in lamellipodia formation via the interaction with clathrin heavy chain (CHC), a protein known to be involved in membrane trafficking. In this new role CHC recruits the WAVE complex to the membrane, increasing the speed of protrusion and cell migration (Gautier et al., 2011). Interestingly, this mechanism is conserved from *Drosophila* until mammalian cells (Kunda et al., 2003; Gautier et al., 2011). Moreover, in another study it was shown that activated ARF1 (ADP-ribosylation factor 1) GTPase triggers the recruitement of AP-1 (Adaptor Protein-1) and clathrin on the trans-Golgi network (TGN) membranes. At the edges of the clathrin-, AP-1 coated subdomains also the CYFIP, ABI, NAP1 complex is recruted. HIP1R binding to clathrin light chains could prevent actin polymerization on the surface of clathrin coats. In a second step, activated Rac1 binds to CYFIP and activates the actin nucleation complex leading to N-WASP-dependent activation of ARP2/3 and actin polymerization toward the TGN membrane. This mechanism provides complementary but independent levels of regulation during early stages of clathrin-AP1-coated carrier biogenesis. Thus the WAVE complex, or at least a CYFIP-containing subcomplex, participates to different clathrin functions (Anitei et al., 2010). Interestingly enough, inactivation of *CYFIP1* in MCF-10A (an immortalized but not transformed mammary epithelial cell line able to form 3D acinar structures in Matrigel) produced acini with abnormal structures while cells expressing normal CYFIP1 levels display a normal morphology. Knockdown of WAVE pathway components, Nckap1 and WAVE 2, generated phenotypes similar to those observed upon CYFIP1 silencing, while inactivation of *FMR1* has no impact on cell morphology. Furthermore, silencing of *CYFIP1* interferes with normal epithelial morphogenesis and cooperates with Ras to produce invasive carcinomas *in vivo* (Silva et al., 2009). A proapoptotic role was also proposed for CYFIP2 by interacting with the Insulin-like growth factor 2 mRNA-binding protein-1 (IMP-1; Mongroo et al., 2011).

Very recently, in fly a new motif, named WIRS, was identified defining a new class of ligands of the WRC including ~120 membrane proteins (e.g., protocadherins, ROBOs, netrin receptors, neuroligins, GPCRs, and channels). The WIRS peptide motif specifically interacts with the surface formed between Sra (CYFIP1/2) and Abi2 (Ortholog of Abi1 and Abi3). In *Drosophila* mutations altering this interaction result in the disruption of actin cytoskeleton organization and egg morphology leading to female sterility (Chen et al., 2014).

CYFIP1 and 2 are localized at the synapse and have been described to interact with FMRP in a GTP-dependent manner. Interestingly, while CYFIP1 interacts only with FMRP, CYFIP2 was also shown to interact with FXR1P and FXR2P, the two paralogs of FMRP belonging to the FXR (Fragile X related) genes family. These two proteins share a high level of homology with FMRP and they are supposed to have a similar function, probably partially compensating for the absence of FMRP in FXS patients (Schenck et al., 2001). CYFIP1/2 are not RNA-binding proteins and their function is thought to modify some functional properties of FMRP. Indeed the domain of CYFIP1/2 interaction with FMRP is the same that mediates homo-heterodimeryzation of the FXR family (Schenck et al., 2001). This suggested binding with CYFIP1/2 can interfere with the ability of FMRP to dimerize with its paralogs FXR1P and FXR2P. Alternatively, CYFIP1/2 could modify FMRP affinity for RNA. A role of CYFIP1 was also proposed as a component of the translational initiation complex interacting with the FMRP/BC1 complex (Napoli et al., 2008). However, the *in vivo* and *in vitro* formation of this complex is controversial (Iacoangeli et al., 2008a,b). A recent study using double FMR1/BC1 KO mice has definitively shown that FMRP and BC1 cannot belong to the same complex even if they are likely to modulate common target RNAs via independent mechanisms (Zhong et al., 2010). Furthermore, since more than 80% of the FMRP pool is located on translating polyribosomes, the role of a putative CYFIP-FMRP containing initiation complex in regulating initiation of translation is very unlikely to have an impact on FMRP function (Corbin et al., 1997; Feng et al., 1997; Khandjian et al., 2004; Stefani et al., 2004; Aschrafi et al., 2005; Darnell et al., 2011). It is also important to underline that the interaction of CYFIP1/2 via FMRP with polyribosomes was shown, suggesting that CYFIP can also modulate the main function of FMRP when it is associated to actively translating polyribosomes (Schenck et al., 2001). In this context, the link between membrane proteins and CYFIP recently described (Chen et al., 2014) may represent an interesting FMRP-dependent regulation of translation *via* external stimuli driven by CYFIP proteins to actively translating polyribosomes.

CYFIP2 mRNA was also reported to be a target of FMRP, that can modulate its expression (Darnell et al., 2011), creating a double link between the two proteins. It is well known that FMRP modulates the expression of proteins that have an effect on the cytoskeleton [e.g., *MAP1B*, *PP2Ac, p0071* (Brown et al., 2001; Castets et al., 2005; Nolze et al., 2013)] suggesting that the link between CYFIP1/2 and the reorganization of the cytoskeleton is two-fold: on one side *via* its participation to the WRC as a regulator of WAVE activity and on the other *via* its interaction with FMRP.

In conclusion, CYFIP role in RNA metabolism through its interaction with FMRP and/or other RNA-binding proteins, as recently proposed (De Rubeis et al., 2013), should be better determined by a large-scale study that is still lacking to date.

## CYFIP ANIMAL MODELS

### DROSOPHILA

The link between CYFIP proteins and other protein/complexes and in particular with the RhoGTPase pathway, pushed us to develop an animal model in *Drosophila* allowing a first analysis of the role of CYFIP in development/maturation of the nervous system. We considered the fly to be a simplified model since only one homolog of the CYFIP family (*dCYFIP*) and only one homolog of the FXR family (*dFMR1*) are present. *dCYFIP* is specifically expressed in the nervous system and interacts biochemically and genetically with *dFMR1* and *dRac*. d*CYFIP *mutations affect axons (growth, guidance, branching) much like mutations in *dFMR1* and in Rho GTPase *dRac1.* *CYFIP*, like the fly *FMRP* and *Rac1* orthologs, plays a pivotal role in the establishment of neuronal connectivity (Schenck et al., 2001). A similar phenotype has been validated for *Cyfip2* (not yet for *Cyfip1*) in zebrafish (Pittman et al., 2010) and rat hippocampal neurons (Kawano et al., 2005). Since neuromuscular junctions (NMJs) share a number of features with central excitatory synapses in vertebrate brain and constitute the best known synaptic plasticity model in *Drosophila*, we analyzed these structures in *dCYFIP* and *dFMR1* mutant flies. In *dCYFIP* mutants, synapse terminals are shorter and display a higher number of buds than in wild-type animals, indicative of impaired synapse growth (Schenck et al., 2001). Loss of *dFMR1* (Zhang et al., 2001) produces a NMJ phenotype that is opposite to that of *dCYFIP *null flies, suggesting an opposite functional role for these two proteins. Furthermore, a co-overexpression of *dCYFIP* partly rescues the *dFMR1* overexpression phenotype (e.g., short synapses) suggesting that *dCYFIP* negatively controls *dFMR1* at the synapse. Finally, using the fly eye to test for genetic interactions, we could order the three molecules within a pathway where dRac1 controls *dCYFIP* that, in turn, regulates *dFMR1* (Schenck et al., 2003). Both the convergent phenotypes and dosage experiments clearly indicated a molecular link between *dFMR1* and the Rho GTPases pathway in neuronal remodeling. We speculated that *CYFIP* proteins may regulate *dFMR* -mediated translational control. Regulation of NMJ development by *dCYFIP* was confirmed by the study of Zhao et al. (2013) Furthermore, these authors performed a detailed analysis of synapses of *dCYFIP* mutants. Using electron microscopy they showed that synaptic vesicles (SVs) are larger in mutants. While the number of SV was unchanged between mutants and wild-type flies, the number of cisternae was elevated in mutants. These abnormalities suggest that dCYFIP may regulate endocytosis and/or vesicle recycling by inhibiting F-actin assembly (Zhao et al., 2013).

Interestingly, inactivation of *Drosophila CYFIP* resulted also in the reduced expression of Kette (Nckap1) and Scar (WAVE) as well as all the other members of the WAVE complex (Bogdan et al., 2004; Schenck et al., 2004; Qurashi et al., 2007; De Rubeis et al., 2013). Inactivation of each member of the WRC has been shown to produce a similar phenotype, in *Drosophila* as well as in cell lines, as it was was shown in HeLa cells (Gautier et al., 2011), in MEF (Dubielecka et al., 2011) and, as already mentioned, in MCF-10A cells (Silva et al., 2009). Collectively these findings demonstrate the tight regulation of the expression of the members of the WAVE complex that is conserved during evolution as well as their function. In conclusion, silencing of each member of the WAVE complex disrupts its function enabling actin polymerization, lamellipodia formation, and cell migration (Eden et al., 2002; Dang et al., 2013).

### ZEBRAFISH

Observations in the fly have been confirmed in zebrafish, where two CYFIP genes are expressed. Pittman et al. (2010) studied the function of Cyfip2 during eye and brain development by analyzing a mutant (*nevermind,* also called *nev*) isolated in a screen for mutations affecting retinotectal axon pathfinding). These authors showed that Cyfip2 is required to mantain positional information by dorsonasal axons as they project through the optic tract and the tectum. The lamination of the eye is disrupted in *nev* mutants, apparently independently of the axon guidance phenotype. Interestingly, these authors addressed the question of the redundancy between *Cyfip1* and *Cyfip2*, but they were unable to show any compensation of* Cyfip1 *in the absence of* Cyfip2* (Pittman et al., 2010).

### MOUSE

*Cyfip1*-null mouse are lethal at the first steps of embryonic development. Bozdagi et al. (2012) decided to analyze haploinsufficiency of *Cyfip1*. They observed that this condition mimics key aspects of the phenotype of *Fmr1* knockout mice. Indeed, in *Cyfip1* heterozygous mice mGluR-dependent LTD was significantly increased in comparison to wild-type mice. In *Cyfip1*+/- mice mGluR-LTD was not affected in the presence of protein synthesis inhibitor (Bozdagi et al., 2012). Unfortunately, these authors did not analyze the presence of audiogenic seizures in Cyfip1 +/- mice, which is the most relevant phenotype in the FXS mouse model and is dependent on the exaggerated activation of mGluR5. Indeed, this phenotype is rescued by treating mice with MPEP (2-Methyl-6-(phenylethynyl)pyridine) an antagonist of mGluR5 (Musumeci et al., 2000; Yan et al., 2005; Thomas et al., 2012). This experiment would be very important to define common actions/pathways of CYFIP1 and FMRP in neuronal function. Behaviorally, *Cyfip1* heterozygous mice showed enhanced extinction of inhibitory avoidance, similarly to *Fmr1* KO mouse, while no differences have been observed in Y-maze and Morris water maze (to detect alterations in working memory and learning and memory ability, respectively; Bozdagi et al., 2012). On the same track, the inactivation of *Cyfip1* in neurons by siRNA generates dendritic spines that are similar to those observed by silencing *Fmr1 (*immature filopodia) even if their number does not appear to be increased (De Rubeis et al., 2013). However, these latter data are in contradiction with literature concerning the WAVE complex, since if the increased level of immaturity of spines is observed by all investigators, it appears that the reduced activity of the WAVE complex results in a reduced number and length of spines. Indeed, in agreement with previous studies in *Drosophila* NMJ (Schenck et al., 2003, 2004; Bogdan et al., 2004; Qurashi et al., 2007), cultured hippocampal neurons obtained from mice lacking WAVE-1 display a 60% reduction of the extent of neurite outgrowth when compared with wild-type as well as 20% reduction of dendritic spines density, and spines appear more immature (Soderling et al., 2007). These mice display a reduced number (-30%) of post-synaptic spines in CA1 hippocampus and these spines made abnormal synaptic contacts; furthermore, the spine head was flattened, with an abnormal content of internal membrane-bound structures (Hazai et al., 2013). In rat hippocampal neurons, inactivation of WAVE or CYFIP1 resulted in a reduced axonal outgrowth (Kawano et al., 2005), as previously shown in the fly (Schenck et al., 2003). Surprisingly, De Rubeis et al. (2013) do not comment on all discrepancies observed between their results and the literature concerning actin remodeling studies. Even more puzzlingly, they wrote “…..Active-Rac1 promotes CYFIP1 recruitment to the WAVE complex and thus actin polymerization.” Indeed, as already mentioned and described by many authors, activated Rac induces the release of CYFIP/NAP/ABI from the WAVE complex that at this point becomes active since the WAVE complex is intrinsically inactive (Cory and Ridley, 2002; Eden et al., 2002; Derivery et al., 2009; **Figure 1**). In the model of De Rubeis et al. (2013) Rac activation should block the WAVE complex instead than activating it!

At this stage we can only conclude that the function of this protein is complex and its implication in different cell pathways is not easy to study. Only in the future the generation of a conditional mouse model for CYFIP1/2 will allow to answer to many questions concerning the role of this family of proteins (and the WRC in a general manner) in neuronal morphology and maturation.

## GENETICS OF CYFIP1 AND 2 GENES AND ASSOCIATED GENETIC PATHOLOGIES

**FIGURE 2:**
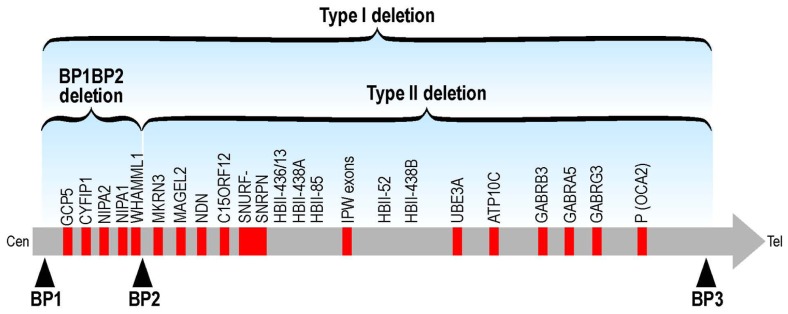
**Schematic representation of TI and TII deletions at the chromosomic region 15q11-q13.** The BP1-BP2 deletion (including *CYFIP1*) is located in 15q11.2.

No point mutations associated to diseases have been described so far in these genes but some indications concerning their impact on human cognition and/or behavior are indicated by genetic abnormalities. Human *CYFIP1* is located in 15q13. Structural abnormalities involving 15q11–q13 are relatively common and many, but not all, of these rearrangements are associated with an abnormal phenotype. Paternal deletions of this region result in Prader–Willi syndrome (PWS) and maternal deletions in Angelman syndrome (AS), both characterized by ID (Cassidy et al., 2000). Interstitial duplications of maternal origin that include the critical region for PWS and AS (PWACR) produce a more variable phenotype, distinct from PWS and AS, that includes hypotonia, ataxia, seizures, developmental delay and autism or atypical autism with no or only minor dysmorphic features. Conversely, paternal duplications of the PWACR are not associated with an abnormal phenotype (Browne et al., 1997). Many different deletions/duplications have been reported to cause these syndromes: class I abnormalities are larger than those of class II since they include four genes (*NIPA1, NIPA2, CYFIP1,* and* TUBGCP5*) and the non-coding mRNA WHAMML1 (Leblond et al., 2012; **Figure 2**). Patients with class I deletions/duplications (TI) seem to have generally more severe behavioral and psychological problems than individuals with class II deletion (TII; **Figure 2**). In PWS, TI deletion also induces an increased cognitive impairment (Buiting, 2010; Peters et al., 2012). For instance in PWS patients carrying the TI deletion adaptative behavior, obsessive-compulsive behaviors, reading, and visual motor integration asseements are in general poorer if compared with PWS patients carrying a TII deletion. Some researchers have analyzed only microdeletions between BP1 and BP2 (Breakpoint 1 and breakpoint 2, respectively – see **Figure 2**). These patients do not have PWS and share several features including different degrees of learning disability, delayed motor and speech development, dysmorphisms and behavioral problems (ADHD, autism, obsessive-compulsive behavior). Two studies reported patients affected by schizophrenia (Stefansson et al., 2008; Kirov et al., 2009) and in one case by epilepsia (De Kovel et al., 2010), while another group recently published a deletion of BP1–BP2 in two young patients affected only by ID and several dysmorphic features (Madrigal et al., 2012). These results suggest that the genes located between the BP1–BP2 breakpoints are determining behavior and intellectual abilities (Bittel et al., 2006). In addition, very recently, BP1–BP2 deletions have been associated to a high risk of dyslexia and dyscalculia (Stefansson et al., 2014). The different authors proposed different conclusions concerning the implication of each gene in the observed phenotype. For instance, Bittel et al. (2006) analyzed the expression level of the four genes in eight PWS patients carrying the BP1 deletion and nine carrying the BP2 deletion and they compared the expression level of each mRNA with the phenotype, concluding that NIPA1, NIPA2, and CYFIP1 may have a greater influence on behavioral and cognitive parameters that have been taken in consideration in this study. In particular NIPA2, a selective Mg^++^ transporter, has the greatest impact, according to these authors (Bittel et al., 2006). Doornbos et al. (2009) proposed that while NIPA1 and CYFIP1 may be important in neurological development and thus play a role in ID and motor/speech delays, TUBGCP5 may have a pivotal role in behavioral abnormalities. Interestingly, TUBGCP5 is ubiquitously expressed with the highest level in subtalamic nuclei, the brain region involved in ADHD and obsessive-compulsive behavior (Doornbos et al., 2009). A reduced expression of mRNA and protein level of CYFIP1 was reported in patients affected by FXS and PWP. These patients displayed ASD, ID associated to hyperphagia and obesity without cytogenetic or methylation abnormalities at 15q11–13 (Nowiki et al., 2007). However, since a genome-wide analysis was not performed on these patients, it is difficult to assess the impact of the perturbed expression of CYFIP1 on their complex phenotype. The findings that in this study ASD occurs in 10 out of 13 patients and autism in 7 out of 13 cases seems to support the implication of CYFIP1 in autism. Conversely, Stefansson et al. (2008) supported the hypothesis that CYFIP1 is involved in schizophrenia due also to its link to FMRP. However, the opinion that “Fragile X behavioral abnormalities resemble features of schizophrenia” appears strongly arguable (Stefansson et al., 2008). Interestingly, recent findings pointed out a common physiopathological mechanism in schizophrenia, autism and ID. Indeed, these studies found an enrichement of mutations causing schizophrenia in genes involved in synaptic pathways that have been already shown to be involved in autism and ID. In some cases, their mRNA is a target of FMRP (Fromer et al., 2014; Purcell et al., 2014). This common etiology of neurodevelopmental disorders could explain also a complex and variable phenotype of deletions of the 15q11–q13 region. However, it wil be important also to perform genome-wide analysis of these patients to assess whether other mutations could contribute to their phenotype.

A submicroscopic chromosome 15q11.2 duplication segregating in a pedigree with ASD was described. By expression analysis of the genes contained in the duplicated region, *CYFIP1 *was suggested to be candidate for involvement in the ASD phenotype in this family. A 30% increase in peripheral blood mRNA levels for the four genes present in the duplicated region in patients, and RNA *in situ* hybridization on mouse embryonic and adult brain sections revealed that two of the four genes, CYFIP1 and NIPA1, were highly expressed in the developing mouse brain (Ingason et al., 2011). These findings point toward a contribution of microduplications at chromosome 15q11.2 to autism, and highlight CYFIP1 and NIPA1 as autism risk genes functioning in axonogenesis and synaptogenesis. Taking into account also its functional properties, CYFIP1 is, among the other genes located in the 15q critical region, the best candidate to produce an ASD (or ID) phenotype when its expression is perturbed. This is consistent with the gene-balance hypothesis, which posits that the same phenotype can arise from under- or over-expression of dosage sensitive proteins because they both disrupt stoichiometry of the same complex (Conrad and Antonarakis, 2007; Darnell et al., 2011) Another example of this situation is provided by findings showing that, as already mentioned, normal synaptic plasticity and cognition occur within an optimal range of metabotropic glutamate-receptor-mediated protein synthesis. In this model, as shown by results in FXS and TSC (tuberous sclerosis), deviation in either direction can cause common behavioral abnormalities (Auerbach et al., 2011).

*CYFIP2* -initially identified as a p53 dependent-apoptosis inducible factor (Saller et al., 1999)- is localized on human chromosome 5q33.3. This gene was not associated to human pathologies so far. Only one case of a girl with a *de novo* deletion of 5q33.3q35.1 affected by psychomotor delay, minor facial anomalies and seizures was described, but we do not know if *CYFIP2* expression was modified by this chromosomal abnormality (Spranger et al., 2000). By homology and analogy with CYFIP1, the function of the two CYFIP proteins may be very similar, as well as their role in neuronal maturation and connectivity.

## Conflict of Interest Statement

The authors declare that the research was conducted in the absence of any commercial or financial relationships that could be construed as a potential conflict of interest.
